# Increased Usage of Antiseptics Is Associated with Reduced Susceptibility in Clinical Isolates of *Staphylococcus aureus*

**DOI:** 10.1128/mBio.00894-18

**Published:** 2018-05-29

**Authors:** Katherine Hardy, Katie Sunnucks, Hannah Gil, Sahida Shabir, Eleftheria Trampari, Peter Hawkey, Mark Webber

**Affiliations:** a Public Health England Birmingham Laboratory, Heart of England NHS Foundation Trust, Birmingham, United Kingdom; b Institute of Microbiology and Infection, University of Birmingham, Birmingham, United Kingdom; c Research and Development, Heart of England NHS Foundation Trust, Birmingham, United Kingdom; d Quadram Institute Bioscience, Norwich, United Kingdom; e University Hospital Birmingham, Birmingham, United Kingdom; f Norwich Medical School, University of East Anglia, Norwich, United Kingdom; McMaster University

**Keywords:** MRSA, chlorhexidine, octenidine

## Abstract

Hospital-acquired infection is a major cause of morbidity and mortality, and regimes to prevent infection are crucial in infection control. These include the decolonization of vulnerable patients with methicillin-resistant Staphylococcus aureus (MRSA) carriage using antiseptics, including chlorhexidine and octenidine. Concern has been raised, however, regarding the possible development of biocide resistance. In this study, we assembled a panel of S. aureus isolates, including isolates collected before the development of chlorhexidine and octenidine and isolates, from a major hospital trust in the United Kingdom during a period when the decolonization regimes were altered. We observed significant increases in the MIC and minimum bactericidal concentration (MBC) of chlorhexidine in isolates from periods of high usage of chlorhexidine. Isolates with increased MICs and MBCs of octenidine rapidly emerged after octenidine was introduced in the trust. There was no apparent cross-resistance between the two biocidal agents. A combination of variable-number tandem repeat (VNTR) analysis, PCR for *qac* genes, and whole-genome sequencing was used to type isolates and examine possible mechanisms of resistance. There was no expansion of a single strain associated with decreased biocide tolerance, and biocide susceptibility did not correlate with carriage of *qac* efflux pump genes. Mutations within the NorA or NorB efflux pumps, previously associated with chlorhexidine export, were identified, however, suggesting that this may be an important mechanism of biocide tolerance. We present evidence that isolates are evolving in the face of biocide challenge in patients and that changes in decolonization regimes are reflected in changes in susceptibility of isolates.

## IMPORTANCE

Infection in hospitals remains a major cause of death and disease. One way in which we combat this is by decolonizing at-risk patients from carriage of bacteria which can cause disease such as MRSA. This is done with antiseptics, including chlorhexidine and octenidine. There is concern, however, that bacteria may be able to become resistant to these antiseptics. In this study, we looked at isolates of MRSA and found that there was a correlation between the use of antiseptics and increased resistance in the isolates. We also suggest that the mechanism by which these more tolerant isolates may become resistant to antiseptics is that of changing a transport pump that exports these agents. This information suggests that we need to study the impact of antiseptics on clinically important bacteria more closely.

## INTRODUCTION

Antiseptics, and especially chlorhexidine, have been used widely as one of the key measures implemented in the control of infections caused by methicillin-resistant Staphylococcus aureus (MRSA) in hospitals. The most widely used approach has been to use antiseptics as part of a decolonization protocol in conjunction with nasal mupirocin for patients who are known to be colonized with MRSA. However, in some units, especially intensive care units, antiseptics have been used universally for washing of all patients (1).

Chlorhexidine, which was first synthesized in 1954 (2), is the most widely used antiseptic. It is a cationic biguanide agent that acts by disrupting the bacterial cell membrane. A more recent introduction to clinical practice is octenidine dihydrochloride, which was synthesized over 30 years later (3). Like chlorhexidine, it is a cationic biguanide agent that has a broad spectrum of activity.

Chlorhexidine and other quaternary ammonium compounds are widely used, being key active components of preservatives and disinfectants (4). They are generally stable, and environmental accumulation is now common, which has led to concerns being raised over the potential for development of resistance (5, 6). Selection of mutants that are resistant to the simpler quaternary ammonium compounds has proved relatively easy under laboratory conditions (7).

Determination of chlorhexidine susceptibility has most often relied on data from MIC determinations. Determination of the MIC is, however, an imperfect method for testing biocide resistance under circumstances in which the lethal rather than the inhibitory concentration of the agent is of primary importance and needs to be measured. In addition, there is no agreed-upon standardized testing methodology and no national or international agreement regarding an appropriate cutoff value for defining chlorhexidine resistance. However, studies have often defined isolates with a chlorhexidine MIC of ≥4 mg/liter as representing resistance (8). No definition has been proposed for octenidine.

The mechanisms of action and resistance to biocides, including chlorhexidine, remain poorly understood, although there are several genes that encode efflux pumps which have been shown to be able to influence biocide susceptibility. Of these, *qacA* is the gene most commonly associated with reduced susceptibility to chlorhexidine in staphylococci. However, the presence of *qacA* does not necessarily result in expression of resistance to chlorhexidine and, conversely, resistance can be expressed without the presence of *qacA* (9–11).

Most studies that have investigated reduced susceptibility to biocides have studied chlorhexidine. The limited numbers of studies that have investigated octenidine have looked at the clinical efficacy and have not included susceptibility testing as part of their studies, with the only *in vitro* study failing to select for resistance to octenidine dihydrochloride (12–14). In contrast to octenidine, reduced susceptibility has been described in chlorhexidine, with the prevalence rates differing between studies (8). Few longitudinal studies have been reported, but one performed in Taiwan observed an increase in the percentage of MRSA strains with reduced susceptibility to chlorhexidine from 1.7% to 46.7% from 1990 to 2005 (15), while significant increases in the chlorhexidine MIC between 1989 and 2000 for both methicillin-susceptible S. aureus (MSSA) isolates and MRSA isolates were observed by Lambert (16). Warren et al. observed a nonlinear increase in the presence of *qacA*- and *qacB* (*qacA*/*B*)-positive isolates, with marked increases in years 5 and 6 of their study but decreases in the subsequent 2 years (17). There have also been case reports of the selection of isolates with an increase in the chlorhexidine MIC in patients receiving chlorhexidine as part of a decolonization regime (18).

Despite the reporting of isolates with reduced antiseptic susceptibility, the clinical impact of this is unclear, with antiseptics being used at much higher concentrations than the typical MIC or minimum bactericidal concentration (MBC) of these isolates. However, there have been reports of clinical failures of decolonization, including both an outbreak of a specific clone of MRSA that had decreased susceptibility to chlorhexidine and the persistence of a hospital clone that carried *qacA* and outcompeted a non-*qacA*-carrying clone (19–21).

This study aimed to investigate whether susceptibility to chlorhexidine and octenidine dihydrochloride, the two biocides most commonly used for the decolonization of patients colonized with MRSA, varied in a unique panel of S. aureus strains isolated over an extended period during which chlorhexidine use increased and octenidine use was introduced (Table 1).

**TABLE 1:** Number of MRSA and MSSA isolates included in each period and corresponding biocide usage

Time period (yrs)	Biocide usage		No. of isolates	
	Chlorhexidine	Octenidine	MSSA	MRSA
1 (1928–1953)	None	None	18	0
2 (1954–2001)	Minimal	None	10	53
3 (2002–2012)	Significant	None	1	47
4 (2013–2014)	Significant	Significant	0	31

## RESULTS

### Usage of chlorhexidine and octenidine.

The use of chlorhexidine and octenidine was analyzed in our hospital, with the results showing a decrease in usage of chlorhexidine and an increase in octenidine use across the study period. The usage of chlorhexidine decreased from 7,061 packs being dispensed in 2009 to 5,091 in 2014. In contrast, the usage of octenidine dihydrochloride increased markedly, with none being prescribed in or before 2013 and 18,844 bottles being prescribed in 2014.

### Biocide susceptibility of isolates.

**FIG 1:**
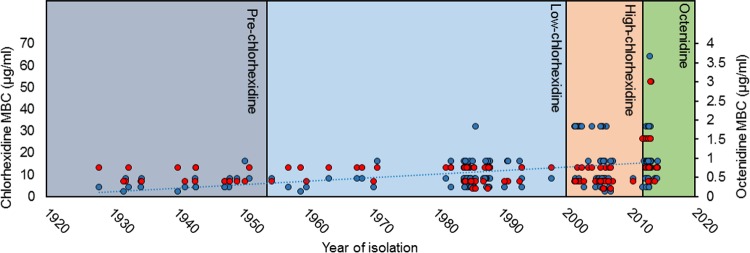
Timeline of mean MBC of chlorhexidine (blue circles) and octenidine (red circles) against isolates. The shaded boxes represent different periods of biocide usage. A trend line (blue, linear) is shown for chlorhexidine but not octenidine, where isolates with decreased susceptibility emerged only in the final period.

**FIG 2:**
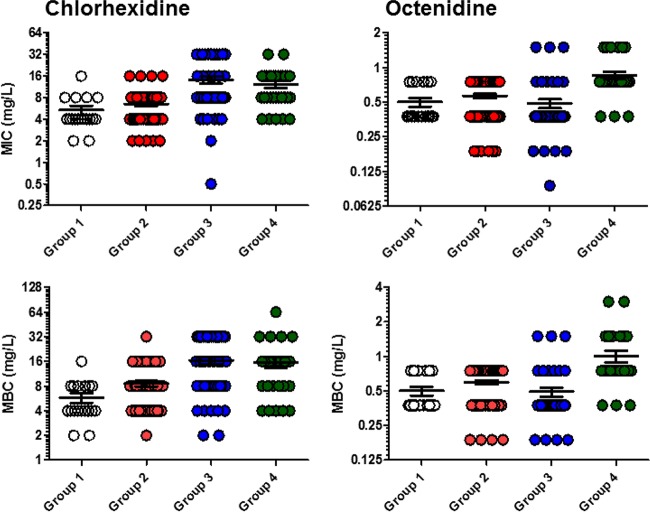
Chlorhexidine and octenidine MIC and MBC values for S. aureus isolates.

The MIC and MBCs of chlorhexidine were significantly different across the four groups (Table 2 and Table 3). Chlorhexidine MICs ranged from 0.5 µg/ml to 32 µg/ml and chlorhexidine MBCs from 2 µg/ml to 64 µg/ml. The mean MIC and MBC of chlorhexidine increased over time from group 1 to group 3 and then plateaued in group 4. The mean MBC of chlorhexidine increased approximately 3-fold (from 5.8 µg/ml to 16.5 µg/ml) between group 1 and group 3 (*P* < 0.0001); group 4 had a slightly lower mean MBC (15.6 µg/ml) than group 3, although the difference was not significant (*P* = 0.78). The increases in mean MIC and MBC values were a result of a shift of susceptibility of most isolates in the population, requiring higher MICs or MBCs, rather than of the presence of a small subpopulation of highly resistant isolates (Fig. 1 and 2). For example, the proportions of isolates with an MBC of >32 µg/ml increased from group 1 to group 2 to group 3 (0% to 1.6% to 31.2% of isolates, respectively) (Fig. 2). The differences between the higher MIC and MBC distributions seen in groups 3 and 4 were not likely to be random according to statistical tests (chi-square and Mann-Whitney tests both returned *P* values of <0.05 in comparisons of both group 3 and group 4 to either group 1 or group 2).

**TABLE 2:** Descriptive statistics for MIC data^a^

Drug	Group	MIC range (mg/liter)	MBC_50_ (mg/liter)	MBC_90_ (mg/liter)	MIC mean (mg/liter) (SE)	Group	Mann-Whitney *P* value of group:			
							1	2	3	4
Chlorhexidine	1	2–16	4	8	5.33 (0.79)	1	NA			
	2	2–16	8	8	6.51 (0.42)	2	0.09	NA		
	3	0.5–32	8	32	14.05 (1.53)	3	**<0.0001**	**<0.0001**	NA	
	4	4–32	8	16	12.26 (1.27)	4	**<0.0001**	**<0.0001**	0.99	NA
										
Octenidine	1	0.375–0.75	0.375	0.75	0.50 (0.04)	1	NA			
	2	0.1875–0.75	0.75	0.75	0.56 (0.03)	2	0.25	NA		
	3	0.009–1.5	0.375	0.75	0.49 (0.04)	3	0.26	0.008	NA	
	4	0.375–1.5	0.75	1.5	0.86 (0.06)	4	**<0.0001**	**<0.0001**	**<0.0001**	NA

a Values indicated in bold represent a *P* value of <0.05. NA, not applicable. ANOVA, *P* < 0.0001 (all groups).

**TABLE 3:** Descriptive statistics for MBC data^a^

Drug	Group	MBC range (mg/liter)	MBC_50_ (mg/liter)	MBC_90_ (mg/liter)	MBC mean (mg/liter) (SE)	Group	Mann-Whitney *P* value of group:			
							1	2	3	4
Chlorhexidine	1	2–16	4	8	5.78 (0.79)	1	NA			
	2	2–32	8	16	8.54 (0.67)	2	**0.024**	NA		
	3	2–32	16	32	16.5 (1.63)	3	**<0.0001**	**0.0001**	NA	
	4	4–64	16	32	15.61 (2.21)	4	**<0.0001**	**0.0006**	0.78	NA
										
Octenidine	1	0.375–0.75	0.375	0.75	0.50 (0.04)	1	NA			
	2	0.1875–0.75	0.75	0.75	0.60 (0.03)	2	0.075	NA		
	3	0.1875–1.5	0.375	0.375	0.49 (0.04)	3	0.296	**0.0009**	NA	
	4	0.375–3	0.75	1.5	1.0 (0.11)	4	**<0.0001**	**<0.0001**	**<0.0001**	NA

a Values indicated in bold represent a *P* value of <0.05. NA, not applicable. ANOVA, *P* < 0.0001 (all groups).

In contrast to the results seen for chlorhexidine, the mean MIC and MBC of octenidine did not rise with time across groups 1 to 3 (the only statistically significant difference seen between these groups was for the mean chlorhexidine MBC for group 3, which was actually somewhat lower than that for group 2). There was, however, a significant rise in the MIC and MBC of octenidine in group 4; for example, the mean octenidine MBC for group 4 (1 µg/ml) was double that for group 3. The increase in MICs and MBCs against isolates in group 4 appeared to be a result of an absence of isolates with low MIC and MBC values, which were present in significant proportions in groups 2 and 3 but absent in group 4, after the introduction of octenidine. As well as the absence of the most susceptible isolates, there was also an emergence of isolates requiring a higher drug MBC (3 µg/ml) than was seen in the previous groups, although three isolates with an MBC of 1.5 µg/ml had been isolated at the end of the study period for group 3 (Fig. 1 and 2). Statistically, both the MIC and MBC distributions for octenidine seen in group 4 were significantly different from those seen with all the other groups (tested by chi-square or Mann-Whitney tests).

Interestingly, there was no correlation between the results with respect to susceptibility to the two agents; i.e., a raised chlorhexidine MIC was not likely to be accompanied by a raised octenidine dihydrochloride MIC in the same isolate (tested using Pearson’s correlation test). The changes in susceptibility to the two agents reflected the usage data, although it was not possible to statistically analyze these changes.

### Typing of isolates.

**FIG 3:**
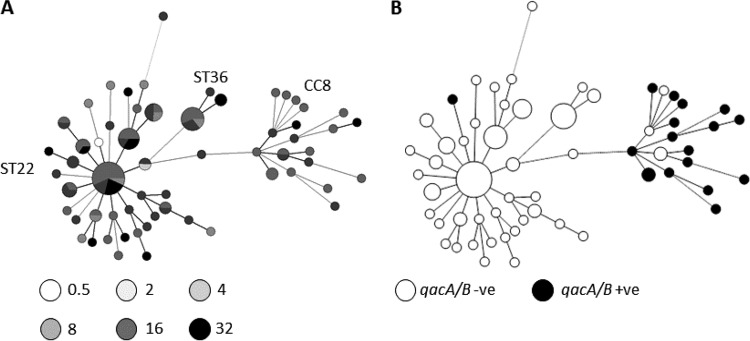
A minimum spanning tree of isolates based on VNTR profiles. Sizes of circles reflect number of isolates. Panel A shows isolates shaded according to the MIC of chlorhexidine (in micrograms per milliliter) per the key below the tree. Panel B shows isolates found to carry *qacA*/*B* (black circles). -ve, negative; +ve, positive.

Variable-number tandem repeat (VNTR) analysis of the isolates revealed clonal complex 22 (CC22), which is the endemic strain within the United Kingdom, to be the most predominant clonal complex. The other two clonal complexes represented were CC36 and CC8 (Fig. 3).

The phylogenetic analysis failed to identify a specific clone or lineage which showed reduced susceptibility to chlorhexidine (Fig. 3). Interestingly, overlaying susceptibility data onto the phylogeny demonstrated several instances where isolates with the same VNTR profile differed in the MICs of both chlorhexidine and octenidine dihydrochloride, suggesting that the acquisition of decreased susceptibility is not restricted to one clonal complex and that it may be able to evolve independently from various strains (Fig. 3).

### Carriage of *qac* genes.

A total of 18/160 (11.3%) of all isolates were positive for carriage of the *qacA*/*B* gene; apart from 1 MSSA isolate, all of these were MRSA isolates. All of the isolates carrying the *qacA*/*B* gene had a chlorhexidine MBC of >4 µg/ml. The majority of isolates in the collection with the highest MICs and MBCs did not, however, carry *qacA*/*B*. The highest proportion of isolates carrying the *qacA*/*B* gene (22%) was in group 2, in contrast to groups 3 and 4, where only 6% and 8% of isolates were carrying *qacA*/*B*. *qacA*/*B* was not detected in any of the pre-1954 isolates in group 1. VNTR typing of all the *qacA*/*B*-positive isolates revealed that all but one of the *qacA*/*B*-positive isolates clustered and belonged to CC8 (Fig. 3). The other isolate was from the sequence type 22 (ST22) cluster, and no isolates from the ST36 cluster carried a *qac* gene.

### Genomic analysis of ST22 isolates.

**FIG 4:**
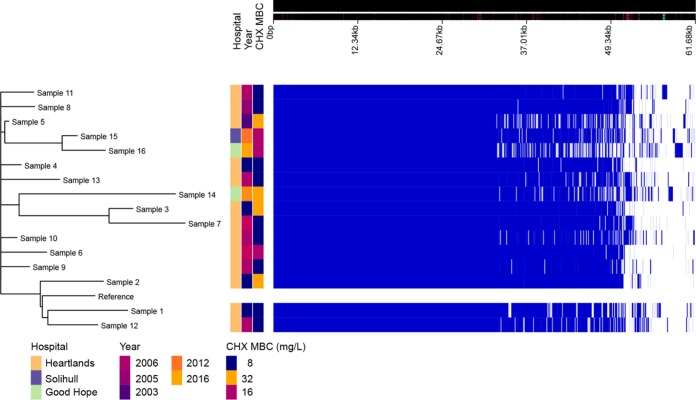
Visualization of pan-genome analysis by Roary of 16 isolates. Hospital and year of isolation are indicated as well as chlorhexidine (CHX) MBC.

Sixteen strains with related VNTR profiles from groups 3 and 4 and a range of chlorhexidine MBCs were subjected to whole-genome sequencing, and mechanisms which may contribute to decreased susceptibility to chlorhexidine were identified. Figure 4 shows the phylogeny of these strains based on a whole-genome alignment (generated using Roary). Comparisons of the accessory genome content, resistance genes (analyzed by ARIBA), and presence and absence of core genes were performed in attempts to identify common changes in those isolates with the highest MBCs. There were no common accessory genes identified, and no carriage of a known resistance mechanism that correlates with biocide resistance was detected. To further try to identify a tolerance mechanism, two pairs of strains that showed 4-fold differences in susceptibility to chlorhexidine (by MBC) but which were very closely related according to the phylogeny data were compared for changes. Strain 7 (MBC of 32 μg/ml) was compared to strain 3 (MBC of 8 μg/ml), and strain 2 (MBC of 32 μg/ml) was compared to the reference ST22 isolate, HO_5096_041 (MBC of 8 μg/ml) (Fig. 4). Analysis of these strain pairs found a small number of single nucleotide polymorphisms (SNPs) in each pair (5 SNPs for the first pair and 20 SNPs for the second pair). The two more resistant isolates both had changes in one of two homologous efflux pump systems, NorA and NorB. Both have been shown to export multiple agents, including biocides (22). Strain 2 carried an SNP within *norB* that resulted in a change of the NorB protein of Met314Ile. This substitution is adjacent to the predicted translocation pore and may alter the capacity of this strain to export chlorhexidine compared to the reference strain. Strain 7 had a wild-type *norB* allele but carried an SNP within *norA* that resulted in a change in NorA of Ala362Val. Substitution at this site has previously been shown to improve the efflux capacity of the protein for some substrates (23). None of the other sequenced strains had changes within either system or in their known regulators.

## DISCUSSION

This study highlighted the finding that the increasing use of chlorhexidine and octenidine dihydrochloride is associated with emergence of reduced susceptibility to each agent in S. aureus. As described in previous studies (15, 24), the number of S. aureus isolates (and especially MRSA isolates) that have demonstrated reduced susceptibility to chlorhexidine has increased over time, which in the current study was most marked when the MRSA epidemic was at its peak within the United Kingdom.

There have only been a limited number of studies that have investigated reduced susceptibility to chlorhexidine longitudinally, and all had examined shorter time periods. Historical isolates included in this study from a period of no antiseptic usage had very low MIC and MBC values, and increases were then observed in groups 2 and 3. Interestingly, in contrast to other longitudinal studies where there have been similar observations, the increases in MIC and MBC values that we observed for chlorhexidine plateaued in the fourth time period. This may reflect the reduction in the number of MRSA infections within the United Kingdom in that most recent time period and the consequent reduction in chlorhexidine usage in our hospital.

We also demonstrated a reduction in susceptibility to octenidine following its universal introduction. There have been a limited number of studies investigating the clinical efficacy of octenidine dihydrochloride, which, while the data were limited, demonstrated efficacy comparable to that of chlorhexidine, but those studies did not include susceptibility testing as part of the analyses (13, 14). There has been very little *in vitro* work investigating the potential development of resistance to octenidine, although one study did attempt to select for resistant mutants (without success) (12).

While there was a demonstrable reduction in susceptibility in isolates seen in the current study and while this markedly occurred after the introduction of octenidine into practice, the MIC and MBC are still relatively low and significantly below the concentrations at which the antiseptics are used in practice. It is unclear what impact these relatively small changes in tolerance as measured *in vitro* have in practice. Comparing the MIC values to the in-use concentrations suggests that it is unlikely that these isolates will affect clinical efficacy, but they have emerged in a real-world situation, suggesting that there is a significant benefit which has been selected in practice. Interestingly, isolates with decreased susceptibility to octenidine dihydrochloride emerged within the first year of its introduction into clinical practice.

For both biocides, isolates with decreased susceptibility were not clonal, which suggests that there has been no emergence of a dominant clone with an advantage in the face of either biocide. The fact that isolates with decreased susceptibility are seen in various strain backgrounds suggests that the capacity to develop this phenotype is common to many strains and presumably results from a change in the S. aureus core genome. This differs from previous reports of the spread of a dominant clone with reduced susceptibility (20).

As highlighted by the review by Harbarth and colleagues, the increased usage of antiseptics has not been matched by an increase in surveillance (both microbiological and clinical) (25). One of the reasons for the lack of surveillance is the lack of both standardized testing methodology and definitions for resistance. The majority of studies have utilized MICs, but the methodologies vary and the technique is less applicable for antiseptics where the lethal concentration rather than the inhibitory concentration needs to be measured and MBC testing is more meaningful. As there is no standardized testing methodology, there has been no national or international agreement regarding an appropriate cutoff value for defining resistance. The clinical impact of the reduced susceptibility demonstrated *in vitro* also needs to be assessed, with the concentrations of antiseptics measured *in vitro* being much lower than the concentrations of antiseptics achieved *in vivo*.

Epidemiological typing in this study revealed that isolates with high MICs are spread across VNTR profiles, both in ST22 (the predominant epidemic clone observed in the United Kingdom) and in CC8. In comparisons performed to detect the presence of *qacA*/*B*, there was no correlation with chlorhexidine susceptibility; the majority of *qacA*/*B* isolates were from CC8. This clonal complex contains ST239, which has previously been described as carrying the *qacA*/*B* genes and has been associated with clinical failure of chlorhexidine (21). Carriage of *qacA*/*B* was seen in only one ST22 isolate in our panel, which is the most predominant ST both within the study hospital and in the United Kingdom. Despite the lack of carriage of *qacA*/*B* in ST22 isolates, the number of isolates with raised MICs was the same as among those from the CC8 group, highlighting further the lack of correlation between the presence of *qacA*/*B* and reduced susceptibility to chlorhexidine.

Consistent with the lack of correlation of *qacAB* carriage and antiseptic susceptibility, a genomic analysis of a subset of strains revealed no common mobile element which could be shown to be associated with chlorhexidine susceptibility. In two pairs of isolates with different chlorhexidine MICs, mutations within chromosomal multidrug efflux systems *norA* and *norB* were found. NorA and NorB have previously been associated with chlorhexidine tolerance for isolates that overexpress these systems, demonstrating decreased chlorhexidine susceptibility (22, 24, 26), although another study reported only a weak association (27). Both *norA* and *norB* are part of the core S. aureus genome, which is consistent with our observation that decreased susceptibility to chlorhexidine can emerge from multiple lineages and is not conditional on horizontal acquisition of *qacA*/*B*. Previous studies have focused on changes in expression of efflux systems as a mechanism to increase the efficiency of export of substrates. Mutation within the structural pump protein of a multidrug efflux system was also recently shown to increase the efficiency of export of some substrates at the expense of others (28). The substitutions within NorB (adjacent to the translocation pore) and NorA (known to alter pump efficiency for fluoroquinolone export) identified here may reflect adaptations to increase the efficiency of chlorhexidine export.

The significance and clinical impact of the emergence of isolates with decreased susceptibility to decolonization regimes remain uncertain. However, our data show that the susceptibility of the studied S. aureus population to antiseptics has shifted over time. It is not possible to demonstrate from these data a causative link between antiseptic use and changes in susceptibility, although differences in antiseptic usage and population sensitivities were seen in conjunction. There is an obvious need for more research in this area to provide better surveillance data from larger populations and geographic regimes and to understand the mechanisms of action and resistance to antiseptics better.

## MATERIALS AND METHODS

A panel of 160 non-methicillin-resistant and methicillin-resistant S. aureus strains isolated between 1928 and 2014 was included in the study (Table 1). This panel included some of the earliest MRSA isolates from the 1960s and 1970s, from the National Collection of Type Cultures (NCTC). All MRSA isolates from 2002 onward were collected from one large NHS Trust hospital in the West Midlands of the United Kingdom. Since 2002, the hospital has had a policy of prescribing a 5-day course of chlorhexidine to decolonize all known MRSA-positive patients. In 2014, universal washing of all patients with octenidine dihydrochloride for the duration of their hospital stay was implemented, with those patients identified as being colonized by or infected with MRSA continuing to receive chlorhexidine.

In this study, both screening and clinical isolates were included. The isolates were grouped into four panels (Table 1) to reflect the usage of octenidine dihydrochloride and chlorhexidine from 1928 to 2014 (groups 1 to 4). The first group comprised 18 MSSA isolates from 1928 to 1953, during which time there was no use of either antiseptic, and provided the historical context for susceptibility of populations unexposed to antiseptics. The second group comprised 53 MRSA isolates and 10 MSSA isolates from 1954 to 2001, during which time there was low usage of chlorhexidine and no usage of octenidine. The third group contained 1 MSSA isolate and 47 MRSA isolates from 2002 to 2012, during which time chlorhexidine usage was high but octenidine was not used. The final group contained 31 MRSA isolates from 2013 to 2014, during which time there was high use of chlorhexidine and octenidine had been introduced. The dominance of MRSA isolates over MSSA isolates reflects routine surveillance practice, where MRSA are actively identified in patients on admission but MSSA are not.

The MICs of each agent were determined using broth microdilution (following recommendations from EUCAST) ranging in concentration from 0.0029 µg/ml to 3 µg/ml for octenidine dihydrochloride and 0.0625 µg/ml to 64 µg/ml for chlorhexidine digluconate. Minimum bactericidal concentrations (MBC) were subsequently determined by inoculation of 10 µl of suspensions (following determinations of MICs) onto LB agar and observation of growth after overnight incubation. The presence of *qacA*/*B* was determined in all samples using multiplex PCR (26).

A panel of 99/160 isolates, including all *qacA*/*B*-positive isolates and all isolates from groups 3 and 4, was epidemiologically typed using variable-number tandem repeat analysis as previously described (29). Genomes of a selection of 16 isolates from groups 3 and 4 that included the main circulating clones were also sequenced using Illumina paired-end sequencing. Reads were then analyzed using the nullarbor pipeline (v1.2 [30]) and a standard virtual machine on the MRC CLIMB framework. Pan-genomes were generated using Roary (v8.0), SNPs were called with Snippy (v3.0), and antibiotic resistance genes and mutations were identified using ARIBA (v2.8.1). Trees were visualized with Phandango. All packages used default parameters unless stated otherwise.

Data were obtained for the number of packs of chlorhexidine and octenidine dihydrochloride issued by the pharmacy in the large teaching NHS Trust hospital from 2008 to 2014, the time period the MRSA isolates were obtained from. Statistical analyses of changes in susceptibility patterns between groups used analysis of variance (ANOVA) (Kruskal-Wallis test), chi-square, and Mann-Whitney tests.
